# Communicative diversity scales with social network structure in bottlenose dolphins

**DOI:** 10.1016/j.isci.2026.117954

**Published:** 2026-10-20

**Authors:** Maia Austin, Laura J. May-Collado

**Affiliations:** 1 University of Vermont, Biology Department, Burlington, VT, USA; 2 Smithsonian Tropical Research Institute, Panamá City, Panamá

**Keywords:** bottlenose dolphin, bioacoustics, animal communication, social complexity, communicative complexity, social network analysis, vocal repertoire, cetacean behavior, whistle diversity

## Abstract

Social relationships can shape the demands placed on animal communication, but different dimensions of social organization may affect signal repertoires in different ways. We investigated whether variation in social network structure covaried with whistle repertoire organization in wild common bottlenose dolphins (*Tursiops truncatus*). We combined photo-identification and concurrent acoustic recordings across two field seasons, quantified group social topology and bond-strength structure, and characterized whistle repertoires using Hill diversity and continuous acoustic-distance metrics. Elastic-net models showed that groups with higher clustering coefficients had lower repertoire diversity, whereas mean and variable bond strength were associated with acoustic-distance structure. These relationships persisted after accounting for predominant behavioral state and group size. Our findings indicate that social cohesion and relationship heterogeneity correspond to distinct dimensions of communicative organization, supporting a multidimensional view of social and vocal complexity in fission-fusion societies.

## Highlights

• Whistle repertoire structure covaries with dolphin social network organization
• Highly clustered groups exhibit lower whistle repertoire diversity
• Bond-strength metrics covary with within-group acoustic distance structure
• Fission-fusion societies exhibit multidimensional social and vocal complexity

## Graphical abstract

**Figure:**
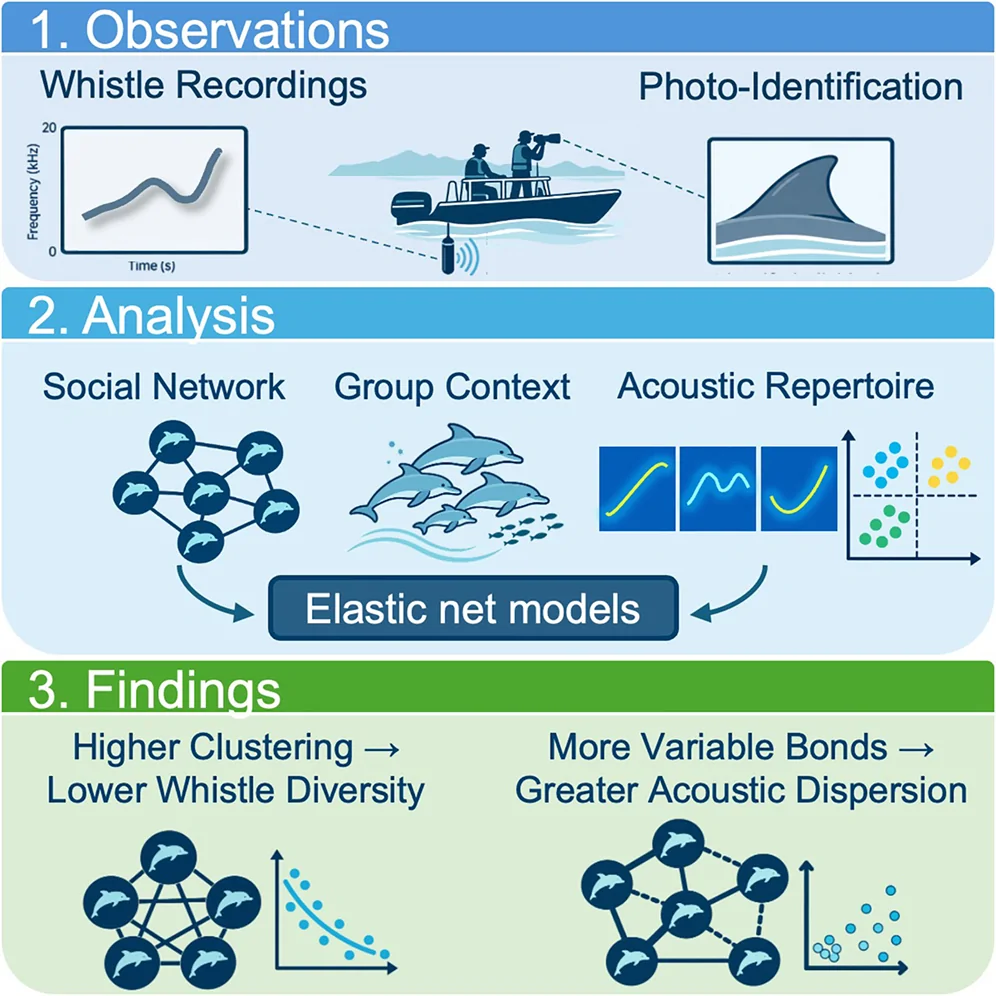

## Teaser

Marine organism; Wildlife behavior; Marine acoustics; Zoology

## Introduction

The evolution of communication is intertwined with the structure and demands of animal social living. Complexity can arise through multiple, non-mutually exclusive mechanisms, including group size and composition, fission-fusion dynamics, role differentiation, and variability in affiliative bonds.^1^^,^^2^^,^^3^^,^^4^ Importantly, social “complexity” may reflect either stability (e.g., enduring differentiated relationships) or flexibility (e.g., dynamic group membership and shifting associations) because both can increase the cognitive and behavioral demands of social living. Accordingly, different features of social organization may represent distinct dimensions of complexity rather than positions along a single continuum. Such demands underpin broader frameworks such as the Machiavellian Intelligence^5^ and Social Brain Hypotheses,^6^ which propose that the challenges of managing dynamic social relationships have driven the expansion of cognitive and neural capacities in many lineages.^7^^,^^8^^,^^9^^,^^10^^,^^11^ Communication, in turn, can both reflect these cognitive capacities and serve as a mechanism through which complex social systems are maintained.^12^^,^^13^^,^^14^

Across taxa, species that live in socially complex groups—those characterized by differentiated, enduring, or hierarchically structured relationships—tend to exhibit correspondingly complex communicative systems.^15^^,^^16^^,^^17^^,^^18^ The Social Complexity Hypothesis for Communicative Complexity (SCHCC) posits that social environments requiring individuals to recognize, track, and coordinate with multiple partners favor the evolution of greater signal diversity, contextual flexibility, and information richness in communication systems.^19^^,^^20^^,^^21^ Evidence supporting the SCHCC has emerged across a wide range of vertebrates, including primates,^3^^,^^22^^,^^23^ bats,^24^ rodents,^25^ and songbirds, including corvids,^12^^,^^26^^,^^27^^,^^28^ suggesting that communicative complexity evolves convergently in response to similar socio-cognitive pressures.

Among mammals, odontocetes (toothed whales, dolphins, and porpoises) provide a particularly powerful system for investigating the links between social and communicative complexity. Many odontocetes live in fission-fusion societies characterized by dynamic group membership, long-term social bonds, and cooperative alliances.^29^^,^^30^^,^^31^ These social features are accompanied by vocal learning—ranging from the individually distinctive “signature whistles” of bottlenose dolphins (*Tursiops* spp.)^32^^,^^33^^,^^34^^,^^35^ to population-level dialects in killer whales^36^^,^^37^ and sperm whales.^30^^,^^38^ Among odontocetes, dolphins show an evolutionary link between the complexity of their tonal acoustic signals—known as whistles—and the size and social organization of their groups, with dolphin species that live in dynamic societies producing highly frequency modulated whistles, which may increase individual distinctiveness and support recognition among frequently changing social partners.^39^ Several dolphin species exhibit individually specific, learned whistles that facilitate social cohesion and recognition within highly fluid group structures.^11^^,^^40^^,^^41^^,^^42^ Therefore, dolphins are well suited for testing the predictions of the SCHCC that communicative complexity scales with social network structure.^23^^,^^43^

Common bottlenose dolphins (*T. truncatus*) produce a diverse acoustic repertoire including tonal whistles and burst-pulse sounds (e.g., buzzes, clicks, squawks, and pops), with whistles broadly divisible into individually distinctive signature whistles and more variable non-signature types. Signature whistles are stable, learned calls functioning primarily in individual recognition and social cohesion,^35^^,^^41^^,^^44^ while non-signature whistles—including graded calls and structurally variable forms—may vary with affective state, group coordination, and social context.^41^^,^^45^ Although burst-pulse sounds play important roles in foraging and close-range social interactions, standardized methods for their cross-individual comparison remain underdeveloped, and we therefore focus on the tonal whistle repertoire. Critically, even in well-studied populations where signature whistles account for roughly half of all vocalizations,^46^ non-signature types vary systematically with behavioral state and social context in ways that signature whistles do not,^45^ providing one potential pathway through which dynamic social context may be reflected in whistle repertoire structure. We therefore characterize the complete whistle repertoire, inclusive of both signature and non-signature types, to capture the full range of acoustic variation available for social modulation.

To test the predictions of the SCHCC, we focused on a small, resident population (>200 individuals) of common bottlenose dolphins inhabiting the archipelago of Bocas del Toro, Panama. This population exhibits high site fidelity and appears to be isolated from other bottlenose dolphin populations in the region, suggesting a relatively closed and demographically small dolphin community.^47^^,^^48^ These characteristics make the population ideal for exploring whether variation in vocal repertoire diversity and structure reflects underlying features of social organization.

Here, we examine the relationship between acoustic diversity (estimated using multiple diversity indices and richness estimators) and social network structure (using several metrics of social group interactions and associations) derived from photo-identification data across two years of field observation of bottlenose dolphins. By linking measures of vocal variation to encounter-level indices of association strength, bond-structure heterogeneity, and group topology, we test whether groups observed in more socially complex group configurations exhibit a more diverse or differentiated whistle repertoire (Table 1; Figure 1). In doing so, we provide a quantitative test of the SCHCC within a wild bottlenose dolphin population, contributing to a growing comparative understanding of animal social communication that connects signaling, cognition, and social structure across taxa.

**Table 1:** Overview of social network and acoustic repertoire metrics, with definitions, interpretation of metric values, and methodological references

Category	Metric	Definition	Significance
Acoustic repertoire	Repertoire richness (q = 0)	Count of distinct whistles within a group^58^^,^^65^	**High:** diverse whistle production with many unique contours **Low:** restricted set of whistles; limited structural variety
	Shannon diversity (q = 1)	Diversity index incorporating both richness and evenness of whistle types or structural variants^28^^,^^58^^,^^66^	**High**: many whistle variants used at relatively similar frequencies. **Low**: a few whistle variants dominate production.
	Simpson diversity (q = 2)	Diversity index emphasizing dominance; lower sensitivity to rare types^28^^,^^58^	**High:** repertoire spread across several frequently used contours. **Low:** repertoire dominated by one or a few recurring contours; structurally stereotyped.
	Mean whistle distance	Mean pairwise dissimilarity among whistles within a group^55^^,^^56^	**High:** group produces diverse contour structures. **Low:** whistles are highly similar; cohesive acoustic profile.
	SD whistle distance	Dispersion in pairwise dissimilarity values^56^	**High:** mixture of similar and divergent whistles. **Low:** uniform whistle contours.
	Range whistle distance	Maximum minus minimum pairwise dissimilarity values within a group^56^	**High**: set of both very similar and very distinct whistles. **Low**: homogeneous whistles
	CV whistle distance	Relative heterogeneity in whistle dissimilarity scaled by mean^56^	**High**: strong variability in contour structure; flexible repertoire usage. **Low**: consistent contour structure across whistles.
Social network	Mean bond strength	Mean of all pairwise association indices among individuals in a group^40^^,^^53^	**High**: cohesive group with strong internal associations. **Low**: weakly bonded group.
	SD bond strength	Dispersion of association strengths within a group^67^^,^^68^	**High**: mixture of strong and weak dyads; socially heterogeneous group. **Low**: uniformly bonded group.
	CV bond strength	Heterogeneity of association patterns relative to typical bond strength^69^	**High**: strong differences in pairwise relationships. **Low**: uniform social structure.
	Group clustering coefficient	Proportion of group members who are mutually connected to each other through shared associations^49^^,^^70^	**High**: tightly cohesive groups; redundant connections **Low**: loose, open network with less redundancy.
	Network density	Proportion of realized edges relative to all possible dyads in the group^49^^,^^70^	**High**: highly interconnected group; widespread associations. **Low**: sparse associations; more fragmented group.
	Degree centralization	Measures whether associations are concentrated around a few individuals^49^^,^^70^	**High**: socially centralized group dominated by key connectors. **Low**: evenly distributed associations.

**Figure 1:**
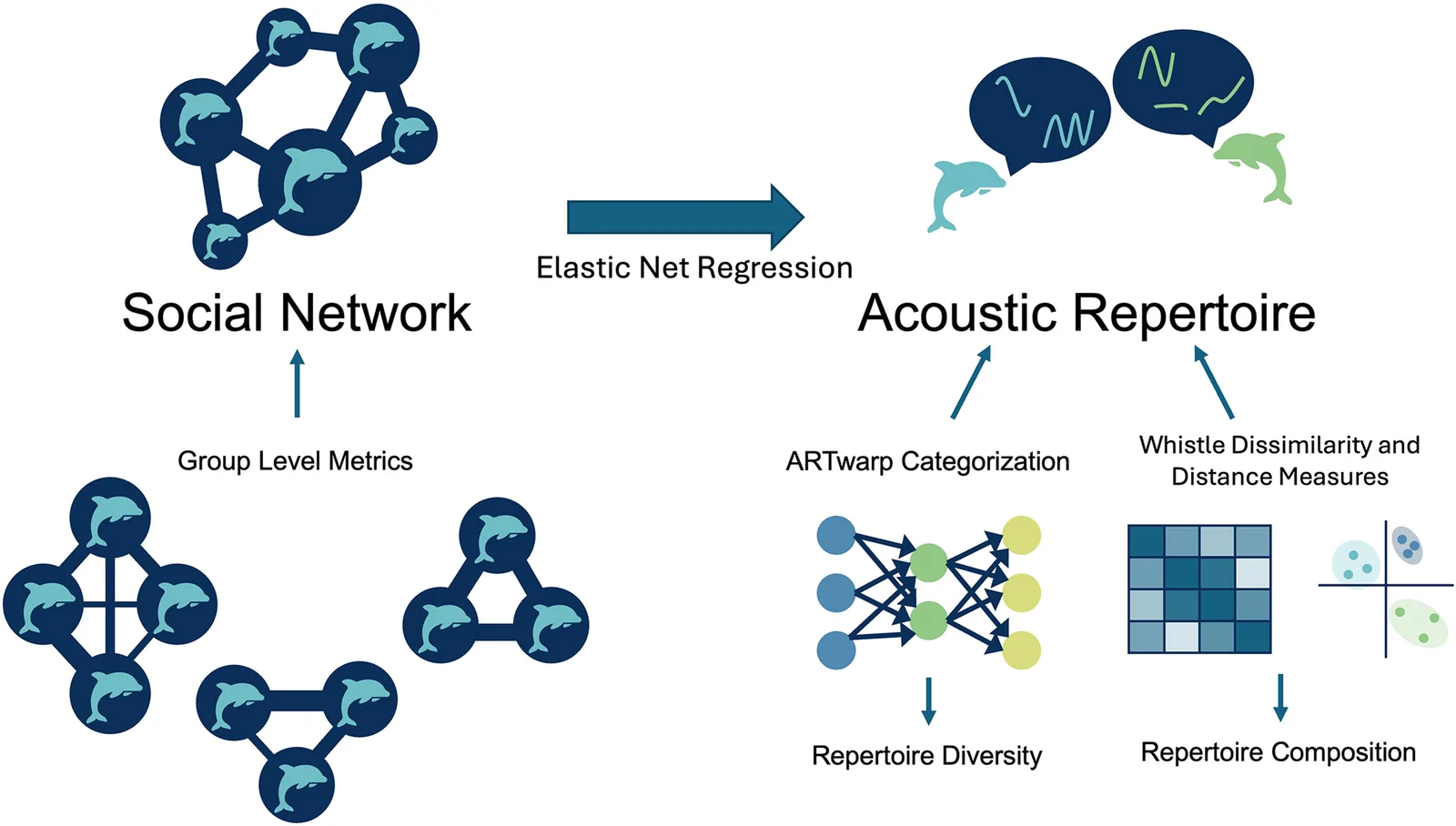
Overview of the workflow used to relate dolphin social networks to acoustic repertoires Social networks were derived from association data and summarized with encounter-level group metrics. Acoustic repertoires were characterized using ARTwarp categorization (diversity) and Luscinia-based dissimilarity matrices (composition). Social and acoustic metrics were integrated via elastic net regression.

## Results

Over the study period, 179 bottlenose dolphin (*T. truncatus*) groups were recorded, and 106 individuals were photo-identified. Capture-recapture estimates based on the Mth Chao model indicated an abundance of 126 ± 15 (SE) individuals, suggesting that approximately 84% of the local population was represented in the photo-ID catalog (AIC = 60.75). After applying filtering criteria requiring sufficient photo-identification coverage and whistle sampling per group, ≥60% photo-identification coverage, no singleton individuals, and ≥2 whistles per identified individual (see STAR Methods), 37 group observations consisting of 72 individuals were retained for analysis. A total of 84.8 h of acoustic recording were conducted, of which 27.3 h corresponded to the 37 filtered group observations included in the final dataset. Across these 37 groups, 3,657 whistles were extracted for analysis (mean ± SD = 151 ± 90.27 whistles per group; range = 51–310), with a mean recording duration of 47.95 ± 15.85 min per group (range = 17.47–81.74).

### Social network structure

Association analyses indicated that the observed dolphin network exhibited non-random social structure. Observed social networks exhibited significantly greater variance and coefficient of variation in association indices than randomized matrices (10,000 data-stream permutations; variance: *p* = 0.001; coefficient of variation: *p* = 0.001), confirming the presence of non-random social preferences and differentiated social relationships within the population. Model comparison using integrated classification likelihood (ICL) supported a three-class mixture structure (ICL = −4444.72), yielding an association complexity (entropy) of 0.889 (Figure 2). Entropy values range from 0 to 1, with higher values indicating greater heterogeneity among latent association classes; thus, the observed value indicates substantial heterogeneity in association structure within the sampled network.

**Figure 2:**
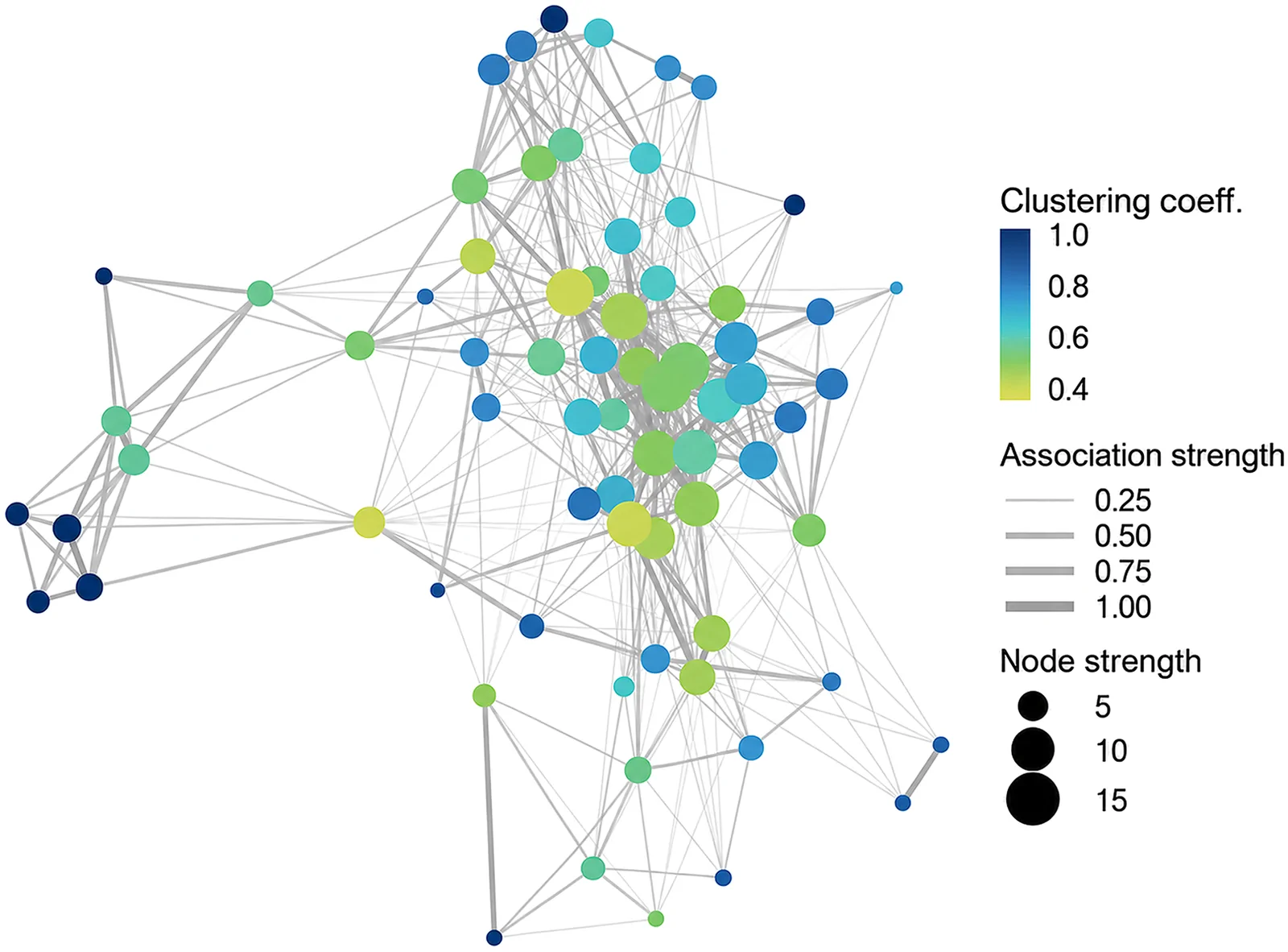
Social network diagram of individuals included in analysis Nodes represent individual dolphins, with node size proportional to individual strength (sum of weighted associations) and node color indicating the local clustering coefficient. Edge width and opacity correspond to the strength of association between individuals calculated using the half-weight index. Half-weight index values range from 0 to 1, where 0 indicates that two individuals were never observed together and 1 indicates that they were always observed together.

Figure 2 shows the social network of individuals observed in the filtered dataset and further illustrates this population’s fission-fusion organization. The network is composed of multiple interconnected clusters with a relatively centralized core and a more diffuse periphery. Individuals in the central cluster display higher node strength but lower clustering coefficients. In contrast, peripheral individuals exhibit higher clustering coefficients and lower node strength.

Group-level social structure varied substantially across sightings (Table S1), including differences in cohesion (group clustering coefficient, and density) and heterogeneity of association strengths (bond strength CV). These metrics capture related but distinct dimensions of encounter-level social structure: clustering and density describe how interconnected group members are, whereas bond-strength metrics describe the magnitude and variability of prior associations among group members. This variation provided the social gradient used to evaluate how group structure predicts whistle repertoire diversity and acoustic distance distributions.

### Social predictors of acoustic repertoire diversity

Elastic net models, which use regularization to select predictors while accounting for collinearity, identified group clustering coefficient as the most consistent predictor of whistle repertoire diversity (Richness, Shannon, and Simpson indices) across groups (Figure 3). Models included predominant behavioral state, group size, calf/juvenile count, and social network metrics under an equal-penalty framework, allowing behavioral, demographic, and social predictors to compete for retention. Groups with higher clustering coefficients—indicating more tightly interconnected association patterns within sightings—exhibited lower repertoire richness and lower Shannon and Simpson diversity (Figures 4A and 4B). Clustering was retained with negative coefficients for richness (β = −0.83), Shannon diversity (β = −0.80), and Simpson diversity (β = −0.21). Socializing was retained with small positive coefficients across richness (β = 0.15), Shannon diversity (β = 0.12), and Simpson diversity (β = 0.12), while resting was retained with small negative coefficients for the same diversity metrics (β = −0.02, −0.01, and −0.01, respectively). Group size, calf and juvenile count, density, centralization, and bond-strength metrics were not retained in the repertoire diversity models.

**Figure 3:**
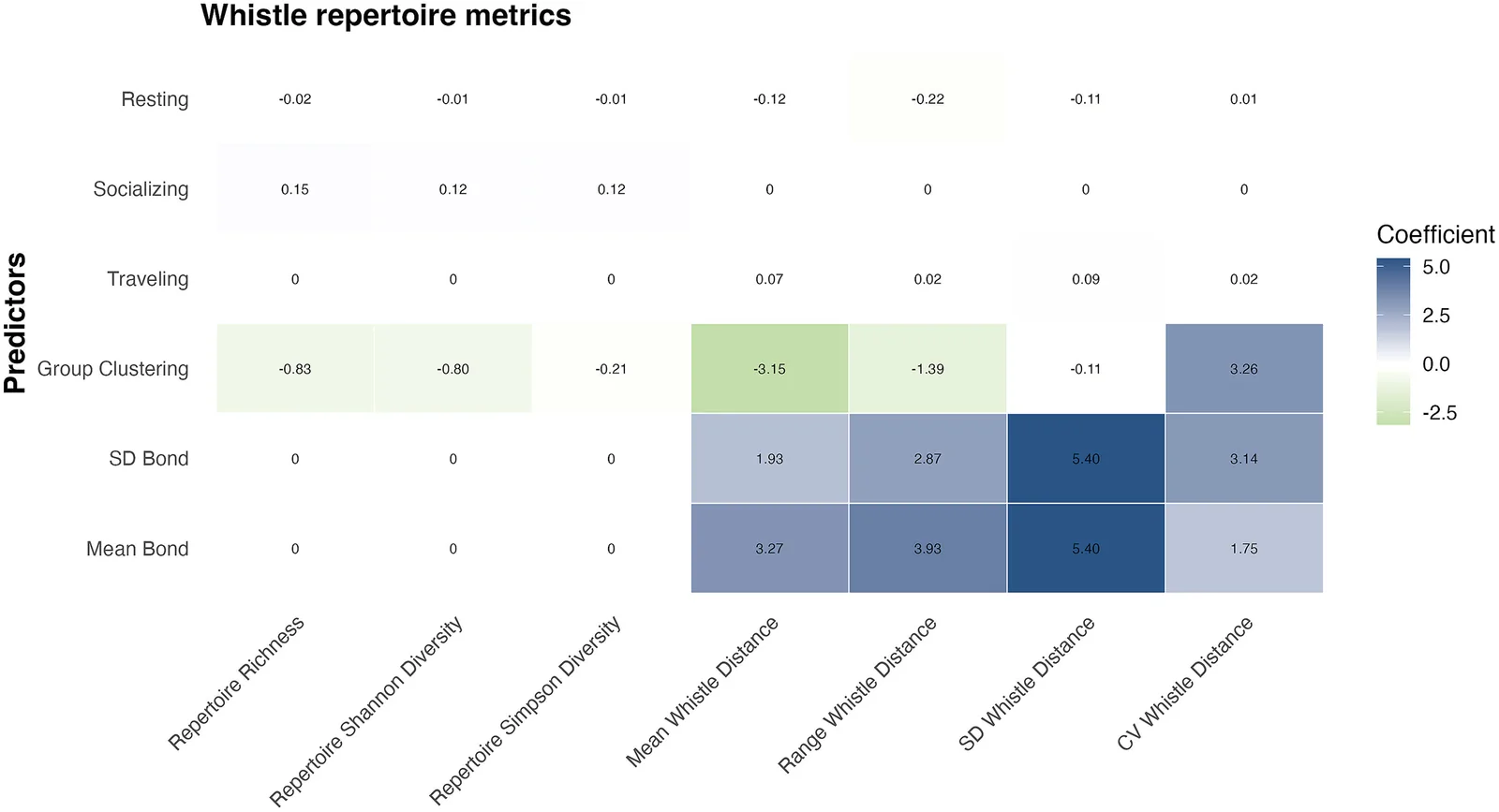
Elastic net model coefficients linking behavioral and social predictors to whistle repertoire metrics Heatmap of standardized coefficients from elastic net regression models predicting whistle repertoire characteristics from predominant behavior, group-level social structure, and demographic covariates. Coefficient values represent standardized elastic-net coefficients at the cross-validated λmin after standardization of continuous predictors and response variables; positive values indicate that higher predictor values were associated with higher response values, whereas negative values indicate inverse associations. White cells indicate coefficients equal to zero, meaning the predictor was not retained for that response after regularization. The color scale reflects the range of retained standardized coefficients across models.

**Figure 4:**
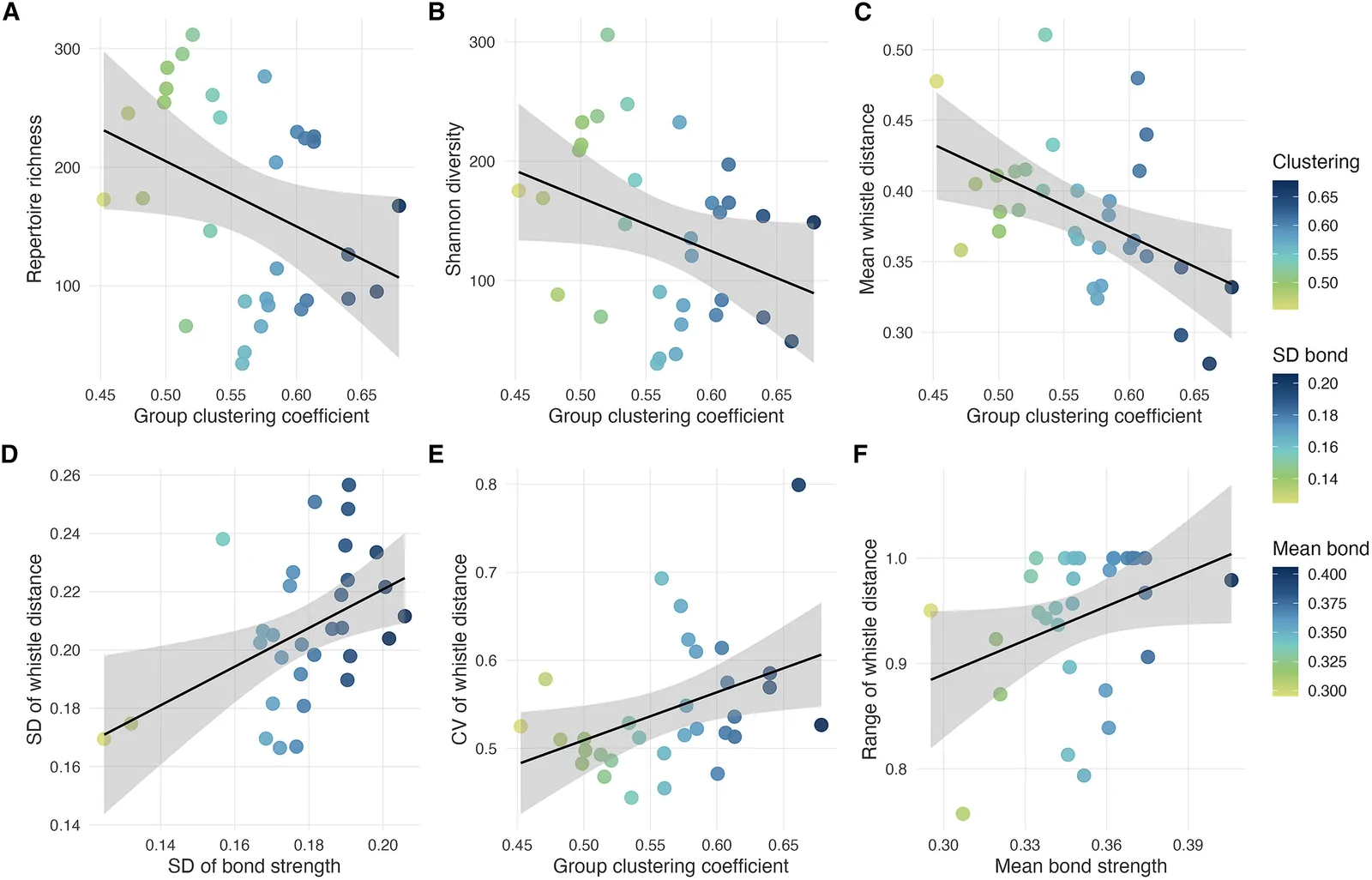
Relationships between group-level social structure and whistle repertoire characteristics (A) Repertoire richness vs. group clustering coefficient. (B) Repertoire Shannon diversity vs. group clustering coefficient. (C) Mean whistle distance vs. group clustering coefficient. (D) SD of whistle distance vs. SD of bond strength. (E) CV of whistle distance vs. group clustering coefficient. (F) Range of whistle distance vs. mean bond strength. Points are colored continuously according to the focal predictor variable, with fitted linear trends (black lines ± SE).

### Social correlates of acoustic distance structure

Elastic net models predicting within-group acoustic dissimilarity retained a consistent set of group-level predictors across distance outcomes (Figure 3). Group clustering coefficient was retained with a negative coefficient for mean whistle distance (β = −3.15), indicating lower average acoustic dissimilarity in more tightly clustered groups, and with a positive coefficient for CV whistle distance (β = 3.26), indicating greater relative variation in pairwise whistle distances after scaling by the mean distance. Group clustering was also retained with smaller negative coefficients for range distance (β = −1.39) and SD distance (β = −0.11).

Bond-strength metrics were repeatedly retained across distance measures, indicating associations between social relationship structure and acoustic dispersion within whistle repertoires. Mean bond strength was retained with positive coefficients for mean distance (β = 3.27), range distance (β = 3.93), SD distance (β = 5.40), and CV distance (β = 1.75). SD bond strength was similarly retained with positive coefficients for mean distance (β = 1.93), range distance (β = 2.87), SD distance (β = 5.40), and CV distance (β = 3.14). In bivariate plots, SD whistle distance increased with SD bond strength (Figure 4D), and range of whistle distance increased with mean bond strength (Figure 4F). Resting and traveling were retained with small coefficients for some distance outcomes, but their magnitudes were smaller than the retained social-network coefficients. Group size, calf/juvenile count, density, centralization, and CV bond strength were not retained in the acoustic-distance models.

To evaluate whether patterns associated with social structure could instead reflect differences in repeated versus rare contour categories, we conducted supplementary models using ARTwarp recurrence metrics as acoustic responses (singleton contours, repeated contours, top-contour dominance, and contour evenness; Figure S1). These recurrence metrics were treated as operational proxies for contour recurrence and stereotypy, not as confirmed signature- or non-signature-whistle classifications, because individual-level whistle attribution was not available. Social and behavioral predictors showed limited and inconsistent associations with recurrence outcomes.

Non-metric multidimensional scaling (NMDS) ordination of whistle similarity provided a multivariate visualization of whistle similarity patterns across groups (Figure 5). The final two-dimensional NMDS solution had moderate stress (stress = 0.131), indicating a usable but approximate representation of the whistle dissimilarity matrix. Whistles were visualized by social clustering tercile (low, medium, and high) to illustrate how acoustic similarity patterns varied across the observed range of group clustering coefficients. Individual whistles are shown as points, with sighting-level centroids and 95% confidence ellipses representing within-group dispersion in the ordination space. When visualized by clustering terciles, whistles from higher-clustering groups occupied more segregated regions of ordination space, whereas whistles from lower-clustering groups showed greater overlap. Because NMDS is a visualization of multivariate dissimilarity, these patterns are interpreted qualitatively and in conjunction with the elastic-net distance models rather than as independent evidence of categorical separation among terciles.

**Figure 5:**
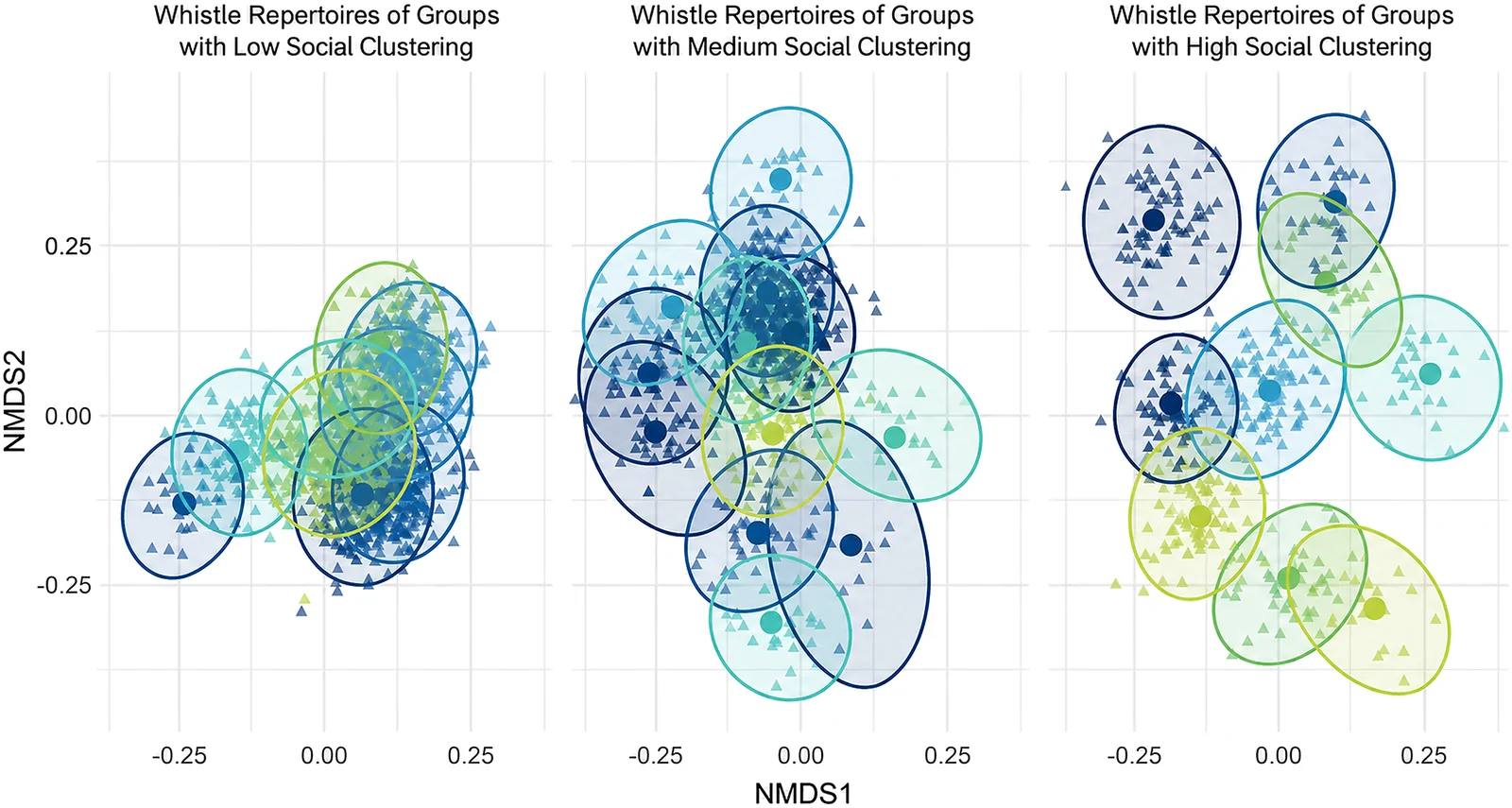
Whistle repertoire similarity across social clustering levels Non-metric multidimensional scaling (NMDS) ordination of whistle similarity among recorded whistles, visualized by group social clustering terciles (low, medium, high). Colors indicate sighting group for the recording; triangles represent individual whistles; circles represent sighting-level centroids; and colored ellipses show 95% confidence regions for groups. The two-dimensional NMDS solution had moderate stress (stress = 0.131).

## Discussion

This study provides quantitative evidence that encounter-level social network structure covaries with whistle repertoire organization in bottlenose dolphins. Across multiple diversity metrics, group clustering coefficient emerged as a consistent predictor of whistle repertoire diversity, with more tightly interconnected groups exhibiting lower richness and diversity of whistle contours. This relationship persisted when predominant behavioral state, group size, and demographic composition were included in the same elastic-net framework, suggesting that the clustering-diversity association was not fully accounted for by broad behavioral context or by the number of individuals present. Together, these results show that variation in encounter-level social structure corresponds with differences in the diversity of whistles expressed within dolphin groups.

In addition to diversity patterns, metrics describing the strength and heterogeneity of social relationships were associated with variation in acoustic distance measures derived from dynamic time-warping comparisons of whistle contours. While bond-structure metrics showed less consistent relationships with categorical diversity indices, they were repeatedly associated with acoustic distance metrics. Groups characterized by greater mean and variable bond strength exhibited broader dispersion in acoustic distance measures, indicating that the structure of social relationships corresponds with increased acoustic differentiation within whistle repertoires. Taken together, these findings indicate that both group cohesion and the distribution of social relationships are associated with multiple dimensions of whistle repertoire structure.

These results emphasize that social complexity is not a single axis.^2^^,^^20^ In this study, group clustering and density capture the degree of cohesion or interconnection among group members, whereas bond-strength metrics capture the magnitude and heterogeneity of prior associations among individuals within a sighting.^49^^,^^50^ The contrasting associations of clustering and retained bond-strength metrics with acoustic outcomes are therefore not necessarily inconsistent with the SCHCC. Rather, they suggest that different dimensions of social complexity may correspond to different dimensions of communicative organization: cohesive, highly interconnected groups may exhibit more constrained whistle diversity, while groups containing stronger or more variable social relationships may exhibit broader acoustic dispersion. This dual-axis interpretation reconciles the reduced diversity observed in highly clustered groups with the broader SCHCC prediction that differentiated and socially demanding relationships are associated with increased communicative complexity. More broadly, these findings suggest that treating social complexity as a single dimension may obscure distinct relationships between social cohesion, relational heterogeneity, and different components of communicative complexity.

Broadly, comparable patterns have been reported within primate populations, where fine-scale social structure predicts within-group variation in vocal behavior. In macaques, individuals embedded in cohesive, high-quality social neighborhoods produce more stereotyped and acoustically constrained calls, whereas callers interacting within more heterogeneous or uncertain local partner sets show greater graded variability and expanded acoustic space.^3^^,^^51^ Likewise, in geladas, stable bonding promotes convergence in call structure, while socially peripheral or weakly bonded individuals exhibit broader acoustic variability.^52^ These intra-population findings parallel the pattern observed here at the group-encounter scale: cohesive topology (high clustering) is associated with reduced repertoire diversity, while bond strength and bond-strength variability are associated with broader acoustic dispersion and expanded within-group distances. Together, these comparisons suggest that communicative complexity may depend not only on the overall number of social partners but also on whether local social structure is cohesive, heterogeneous, or both.

Comparable dynamics are also evident across bottlenose dolphin species, although prior work has focused on different levels of vocal behavior and social context. In Indo-Pacific bottlenose dolphins (*T. aduncus*), variation in vocal usage—such as signature-whistle exchange rate (individual contact calls) and “pop” calls (coercive calls used to hear estrous females)—varies with male alliance structure and social bond strength. Allied males with stronger social bonds exhibit a higher rate of pop call synchrony during cooperative behaviors, while signature-whistle exchange rates are higher among allies with weaker social bonds and fewer direct affiliative contacts.^40^^,^^43^^,^^53^ These studies demonstrate that the use of acoustic signals varies significantly according to social context and the social relationships. This study adds a broader dimension and complements this male alliance-focused work by examining whistle repertoire-level organization and showing that encounter-level topology covaries with both categorical whistle diversity and continuous acoustic distance structure. Importantly, *T. truncatus* males generally do not appear to form higher levels of alliances as those described in Indo-Pacific bottlenose dolphins, although stable male pairs have been reported.^54^ In Bocas del Toro, there is evidence of male pair formation, but the social dynamics of these associations have not been studied yet. Therefore, we interpret the lower diversity observed in highly clustered groups as a general pattern of reduced encounter-level whistle variation in tightly interconnected groups, rather than as evidence for alliance-specific signal convergence.

The NMDS ordination provides a multivariate visualization of whistle similarity patterns across sightings and offers a qualitative complement to the elastic net results.^55^^,^^56^^,^^57^ The final two-dimensional solution had moderate stress (stress = 0.131), indicating a usable but approximate representation of the whistle dissimilarity matrix. When visualized by clustering terciles, higher-clustering sightings appeared to occupy more peripheral and segregated regions of ordination space, whereas lower-clustering sightings overlapped more extensively in a shared central region.^58^ This pattern is congruent with the retained associations between clustering and both categorical diversity indices and distance structure and is consistent with the interpretation that encounter-level cohesion covaries with how whistle pools are distributed in acoustic space. Multiple non-exclusive processes could contribute to this pattern, including shifts in the relative contribution of individually distinctive contours and other context-linked whistle forms that structure bottlenose dolphin repertoires.^35^^,^^41^^,^^42^^,^^59^

Viewed through a cognitive lens, these results map onto expectations from the Social Brain and Machiavellian Intelligence hypotheses.^6^^,^^8^^,^^60^ Socially heterogeneous groups may impose greater informational and cognitive demands: individuals may need to track variable partners, shifting associations, and inconsistent interaction histories. Broader and more even repertoires may reflect communicative flexibility associated with the cognitive demands present in such social environments. Conversely, high-clustering groups may provide predictability and reduce cognitive uncertainty. Vocal use of a more constrained acoustic space may reflect cognitively efficient strategies in more cohesive or predictable social contexts, where expectations and outcomes are more consistent.

These contrasting dynamics are consistent with broader theoretical links among social structure, communicative complexity, and cognition. As social networks become more differentiated, communicative demands may increase and favor expanded signaling repertoires; in turn, flexible communication may facilitate navigation of complex social environments. Such reciprocal processes are central to theories of cultural and communicative coevolution^27^^,^^61^ and may help explain the exceptional vocal plasticity and social learning capacity of delphinids.^62^^,^^63^ The coexistence of more constrained and more dispersed acoustic patterns observed here—associated with different dimensions of social structure—mirrors the dual forces of conformity and innovation thought to underpin culturally transmitted communication systems.

In conclusion, these findings demonstrate that whistle repertoire structure covaries with encounter-level social network organization in bottlenose dolphins. Groups with high clustering exhibited lower whistle repertoire diversity, while bond-strength metrics were repeatedly associated with acoustic distance structure. These relationships persisted after incorporating predominant behavioral state, group size, and demographic composition, indicating that social topology contributes to whistle repertoire organization beyond broad behavioral and demographic contexts. Overall, the results provide a quantitative link between fine-scale encounter structure and whistle repertoire organization in a fission-fusion delphinid system, consistent with broader frameworks that relate social differentiation to communicative complexity.^16^^,^^18^^,^^20^

### Limitations of the study

Because this study was observational and conducted at the group-encounter level, the reported associations should not be interpreted as causal. The final dataset contained 37 group observations, which limited our ability to evaluate interactions among social, demographic, and behavioral predictors. Sex was known for only a subset of the identified dolphins and therefore was not included as a predictor. Consequently, potential sex-related differences in social association patterns or whistle repertoire organization could not be evaluated, which may limit the generalizability of the results. Several non-exclusive mechanisms could also contribute to the observed covariation between social topology and whistle repertoire. First, behavioral state may influence whistle production rates, affective content, or the prevalence of particular whistle forms.^45^ Although predominant behavioral state was included in the elastic-net models, behavioral state was summarized at the group-encounter level, and finer-scale behavioral data from tags, drones, or individual focal follows would be needed to fully separate social and behavioral effects. Second, encounter-level repertoire summaries may be influenced by uneven whistle contributions across individuals and contexts, such that a small subset of highly active callers could disproportionately shape the whistle pool observed during a sighting.^13^^,^^14^^,^^45^ Third, because individual-level whistle attribution was not available, variation in the signature-whistle composition of groups—including differences in the number, identity, or acoustic similarity of signature whistles represented—could not be separated from broader changes in whistle repertoire structure. These factors are not mutually exclusive, and each could contribute to how encounter-level social structure is reflected in the group-level whistle repertoire.

Analyzing the complete whistle repertoire captured both signature and non-signature whistles but introduced an important interpretive limitation: groups with more individuals may appear more diverse simply because more individually distinctive whistle contours are present, while highly clustered groups may appear less diverse if they contain more repeated or acoustically similar stereotyped contours. Supplementary recurrence metrics did not reproduce the main social-network pattern, but recurrence alone cannot distinguish whether reduced repertoire diversity in highly clustered groups reflects differences in signature-whistle composition or socially associated changes in the use of more flexible non-signature whistles. Future work incorporating individual-level whistle attribution will therefore be needed to separate the effects of signature-whistle composition, non-signature-whistle use, and variation in caller contributions.

Sampling was limited to May and June of 2023 and 2024 and to a single resident population, constraining our ability to evaluate seasonal variation and determine whether these patterns generalize to populations with different social or ecological conditions. Although Bocas del Toro does not have clearly delineated wet and dry seasons, and previous work indicates high site fidelity, male and female philopatry, and year-round availability of coastal prey,^47^^,^^64^ unmeasured seasonal variation in prey distribution, habitat use, group composition, or dolphin-watching pressure could influence both social and acoustic patterns. Future year-round sampling could evaluate whether seasonal variation in prey availability, distribution, or dolphin-watching pressure could alter relationships between social topology and whistle repertoire organization. Comparative studies across populations will also be needed to assess the generality of these relationships.

## Resource availability

### Lead contact

Further information and requests for resources should be directed to and will be fulfilled by the lead contact, Maia Austin (austinmaia@gmail.com).

### Materials availability

This study did not generate new unique reagents or materials.

### Data and code availability

• Data reported in this paper will be shared by the lead contact upon request.
• All original analytical scripts used for data filtering, social-network computation, acoustic-diversity estimation, and model fitting are publicly available on GitHub at https://github.com/austinmaia/bocas_schcc.
• Additional information required to reanalyze the data reported in this paper is available from the lead contact upon reasonable request.

## Acknowledgments

This work was supported by a Smithsonian Tropical Research Institute Short-Term Fellowship, a UVM Dr. Roberto Fabri Fialho Research Award, and a John Wheeler Award to M.A.; and by NSF-DEB-2335991 to L.J.M.-C. We thank Dr. Eric A. Ramos, Dr. Betzi Perez Ortega, Megan O’Connor, and Raquel Cardenas for helping to collect data in the field. We are grateful to the Bocas del Toro STRI Field Station team, especially Dr. Rachel Collin, Urania González, and Plinio Góndola, as well as our boat captains, Rene Georget and Demetrio Georget, for their support and guidance in conducting field work.

## Author contributions

Conceptualization, M.A. and L.J.M.-C.; methodology, M.A. and L.J.M.-C.; software, M.A.; formal analysis, M.A.; investigation, M.A.; data curation, M.A.; visualization, M.A.; writing – original draft, M.A.; writing – review and editing, M.A. and L.J.M.-C.; supervision, L.J.M.-C.; project administration, M.A. and L.J.M.-C.; resources, L.J.M.-C.; funding acquisition, M.A. and L.J.M.-C. Both authors approved the final version of the manuscript.

## Declaration of interests

The authors declare no competing interests.

## STAR★Methods

### Key resources table

**Table:** 

REAGENT or RESOURCE	SOURCE	IDENTIFIER
**Experimental models: Organisms/strains**		

Common bottlenose dolphin (Tursiops truncatus), free-ranging Bocas del Toro population	Wild population observed *in situ*, Bocas del Toro Archipelago, Panama	N/A

**Software and algorithms**		

FlukeBook	FlukeBook	flukebook.org/
SOCPROG 2.9	Whitehead Lab, Dalhousie University	whitelab.biology.dal.ca/SOCPROG.html
Avisoft-RECORDER (version used not specified)	Avisoft Bioacoustics	avisoft.com/downloads/
Raven Pro 1.6	Cornell Lab of Ornithology	ravensoundsoftware.com/software/raven-pro/
Luscinia 2.16	Luscinia project	sourceforge.net/projects/luscinia/
MATLAB 2015b	MathWorks	mathworks.com/products/MATLAB.html
ARTwarp v1.0	Zenodo	https://doi.org/10.5281/zenodo.7713615
R 4.3.3	R Foundation	r-project.org/
igraph for R v2.2.1	igraph project/Zenodo	https://doi.org/10.5281/zenodo.7682609
glmnet	CRAN	cran.r-project.org/package=glmnet
vegan	CRAN	cran.r-project.org/package=vegan
Rcapture	CRAN	cran.r-project.org/package=Rcapture
Custom R and MATLAB analysis scripts	This paper	github.com/austinmaia/bocas_schcc

**Other**		

CR3 hydrophone (−207 dB re 1 V/μPa)	Cetacean Research Technology	cetaceanresearch.com/hydrophones/cr3-hydrophone/
UltraSoundGate 416H, 4-channel recording interface	Avisoft Bioacoustics	avisoft.com/ultrasoundgate/416h/

### Experimental model and study participant details

This study examined free-ranging common bottlenose dolphins (*Tursiops truncatus*) inhabiting the Bocas del Toro Archipelago on the Caribbean coast of Panama. Laboratory strain and genotype classifications were not applicable. Observed groups included adults, juveniles, and calves, although exact chronological ages were not known. The number of calves and juveniles in each group was included as a demographic predictor in the analyses. Calves were included in group counts and treated as potential contributors to acoustic diversity but were excluded from individual photo-identification coverage estimates because their dorsal fins were generally insufficiently marked for reliable identification. Sex was known for only a subset of identified dolphins and was therefore not included as a predictor. Consequently, potential sex-related differences in social association patterns or whistle repertoire organization could not be evaluated; this constraint is acknowledged in the limitations of the study section.

Animals were observed and acoustically recorded from vessels using non-invasive photo-identification and passive acoustic methods. No animals were captured, handled, restrained, housed, or experimentally manipulated; therefore, housing, husbandry, and maintenance conditions were not applicable.

All research was conducted under permit ARB-035-2023 issued by the Panamanian Ministry of the Environment (MiAmbiente). All procedures were reviewed and approved by the University of Vermont Institutional Animal Care and Use Committee (IACUC protocol IPROTO202300000018) and the Smithsonian Tropical Research Institute Institutional Animal Care and Use Committee (STRI IACUC protocol SI-25025) and conformed to applicable Panamanian regulations and institutional guidelines for research involving wild marine mammals.

### Method details

#### Study area and field surveys

Data were collected on bottlenose dolphins (*Tursiops truncatus*) inhabiting the Bocas del Toro Archipelago, Caribbean coast of Panama (9°20′N, 82°15′W). This region is characterized by shallow embayments, mangrove islands, and coastal channels with high levels of boat traffic and dolphin–watching tourism.^48^^,^^71^^,^^72^ Surveys were conducted during May and June of 2023 and 2024 (Figure S1).

Boat–based surveys were conducted from small outboard–powered vessels operating 7–10 h per day, during good weather conditions (Beaufort wind scale ≤3), for a total of 28 survey days in 2023 and 14 in 2024. Effort followed established routes through the Dolphin Bay and Almirante Bay regions, maintaining a consistent observation distance within ≤200 m of focal groups.

#### Group definition and behavior

Groups were defined using the 10-meter chain rule, in which any dolphin within 10 m of another group member was considered part of the same group.^73^ During each sighting, the group activity (foraging, socializing, traveling, resting, or milling) was recorded at regular intervals (5 min) and summarized as the predominant behavioral state across the encounter. Activity states followed standard definitions: traveling (persistent, directional movement), foraging (prey pursuit or repeated re-orienting consistent with feeding), socializing (physical contact, chases, or socio-sexual behavior), resting (slow movement with low surface activity), and milling (non-directional movement without clear foraging or social activity). Predominant behavioral state was subsequently included as a categorical predictor in the elastic-net models and was interpreted as a broad encounter-level measure of behavioral context rather than a measure of individual-level behavior. Calves were included in group counts and treated as potential contributors to acoustic diversity in modeling but were excluded from individual identification coverage estimates due to the difficulty of reliably identifying individuals with unmarked dorsal fins.

#### Photo-identification and abundance

High–resolution photographs of dorsal fins were collected using DSLR cameras and later processed using FlukeBook (www.flukebook.org) for automated fin detection and matching. All identifications were manually validated. To estimate total population size and sampling coverage, we applied closed–population capture–recapture models in the R package Rcapture.^74^ Daily encounter histories were used as sampling periods. A closed framework was appropriate given the shorter sampling period and the population’s strong site fidelity and limited short-term demographic turnover.^48^ We fitted standard closed-population models (M0, Mt, Mh, and Mth) and selected among them using AIC. The Mth Chao estimator provided the best fit and was biologically appropriate, as it accounts for both temporal variation and individual heterogeneity in capture probability, given differences in survey conditions, group size, and mark distinctiveness. Abundance estimates, standard errors, and sample coverage were extracted from this top-ranked model.

#### Acoustic recording

Acoustic data were collected concurrently with behavioral observations using four CR3 hydrophones (−207 dB re 1 V/μPa; Cetacean Research Technology, Seattle, WA) connected to a four–channel Avisoft UltrasoundGate 416H interface. Recordings were made in Avisoft–RECORDER at a sampling rate of 187.5 kHz, with hydrophones deployed at a depth of 2 m. The vessel was repositioned as needed to maintain a 200–500 m range from the group while minimizing engine and handling noise. Recordings were assigned to the focal group only when no other dolphin group was visually detected within acoustic range during the encounter; recordings with ambiguous group attribution were excluded from analysis.

#### Social network construction and metrics

Social association networks were constructed in SOCPROG 2.9^4^ using the half–weight index (HWI) to quantify pairwise association strength based on co–occurrence across daily sighting events. A one–day sampling period was used to reflect the population’s fission–fusion dynamics while minimizing pseudoreplication. The HWI accounts for missed identifications and partial group sampling by giving reduced weight to sampling occasions in which only one member of a dyad is identified, yielding values from 0 (never associated) to 1 (always associated).

#### Network randomization and complexity

To evaluate whether the observed network structure departed from random expectations, we performed permutation tests based on SOCPROG’s “data-stream” randomization procedure.^4^^,^^75^ This procedure permutes group membership while preserving sighting structure and sampling effort, generating null distributions for variance and coefficient of variation in association indices. Observed metrics were compared to 10,000 randomized networks, and significance was assessed using two-tailed permutation *p*-values.

The structural complexity of the full association network was quantified using a binomial mixture model fitted to the association matrix following Whitehead.^4^ Mixture models with different numbers of latent classes were compared using the ICL, which balances model fit and clustering uncertainty. The model with the lowest ICL was selected, and the corresponding entropy value was used as an index of association heterogeneity, with higher entropy indicating a greater diversity of underlying association types.

Group-structural metrics were calculated in R using the igraph package.^76^ For each sighting’s induced subgraph, we computed the mean, standard deviation (SD), and coefficient of variation (CV) of bond strength, where bond strength represents the HWI values between all pairs of individuals identified within the sighting. These metrics describe not only the magnitude of associations among group members but also the heterogeneity of those relationships. We also calculated group clustering coefficient, which reflects the overall triadic closure within the subgraph. Network density (the proportion of realized associations relative to all possible dyads) and degree centralization (the extent to which associations were unevenly distributed across individuals) were also computed for each group. Taken together, the network metrics quantify encounter-level association topology and bond-strength structure within each sighting subgraph.

#### Acoustic signal processing

All acoustic files were visually screened in Raven Pro 1.6 with a fast Fourier transform (FFT) size of 2,048 points, 50% overlap (256-sample hop), and a 512-sample Hann window, corresponding to a frequency-bin spacing of approximately 91.6 Hz at the 187.5-kHz sampling rate. Candidate whistles were retained only when the fundamental frequency contour was visually continuous, non-overlapping with other strong signals, and sufficiently visible above background noise to allow consistent manual tracing in Luscinia. Candidate whistles were manually detected from spectrograms by trained observers experienced in dolphin acoustic analysis. All detections and contour traces were subsequently reviewed and validated by MA prior to inclusion in the final dataset. Acoustic composition metrics were derived from pairwise whistle dissimilarities generated in Luscinia.^55^^,^^57^ Fundamental frequency contours were extracted manually by tracing each whistle using Luscinia’s dynamic range tool, which adjusts grayscale contrast to ensure that only signal components with sufficient signal-to-noise ratio are included. For each extracted contour, Luscinia calculates a suite of spectrographic measurements describing the temporal and frequency structure of signals, including instantaneous frequency values along the contour, contour duration, minimum and maximum frequencies, and local slopes. The acoustic dataset included all tonal whistle contours that met quality criteria. We did not assign whistles to signature or non-signature classes because individual-level acoustic attribution was not available. Burst-pulse sounds, echolocation clicks, buzzes, pops, and other broadband or pulsed signals were excluded because the present analyses were designed around contour-based comparison of tonal whistles.

Contours were then measured and processed within Luscinia 2.16 using two complementary pipelines. First, contours were directly compared in Luscinia with dynamic time warping (DTW) to produce a pairwise whistle dissimilarity matrix.^55^ DTW aligns time–frequency trajectories and yields a continuous distance metric that captures graded variation among whistles while accommodating temporal stretching and compression; the resulting dissimilarity matrix was used for multivariate composition analyses (NMDS) and for calculation of distributional summary statistics (mean, SD, CV, and range) of within group acoustic distances.

Second, we extracted peak frequency contours in Luscinia and exported them as.ctr files via a custom MATLAB script. This MATLAB export routine standardized contour formatting. Exported.ctr files were then fed to ARTwarp,^77^ an automated contour-clustering approach combining adaptive resonance templates with DTW, producing discrete whistle categories for use in repertoire diversity indices. These two streams are complementary: the DTW/dissimilarity pipeline preserves continuous, “fuzzy” comparisons of acoustic structure for composition and spread analyses, while the ARTwarp stream produces repeatable categorical units for richness estimation and coverage–standardized diversity metrics.^58^ Together they provide a robust, complementary characterization of group-level whistle repertoire structure.

#### Acoustic diversity metrics

Acoustic diversity within each sighting group was quantified using two complementary approaches: (1) Hill numbers to capture repertoire diversity and (2) acoustic composition metrics derived from pairwise whistle dissimilarities in Luscinia.

Hill numbers provide a unified framework for estimating repertoire diversity while accounting for the relative abundance of different whistle types.^78^^,^^79^ Under this framework, diversity can be expressed at different orders that weight rare and common signal types differently.^80^ In this study, we calculated repertoire richness (Hill number q = 0), Shannon diversity (Hill number q = 1), and Simpson diversity (Hill number q = 2). Richness weights all signal types equally, Shannon diversity incorporates both richness and evenness by weighting common signal types more strongly, and Simpson diversity emphasizes dominant signal types. When combined, these measures provide a robust characterization of both repertoire breadth and evenness.^58^

To evaluate whether social-acoustic patterns could be explained by differences in repeated versus rare whistle contour categories, we derived supplementary ARTwarp recurrence metrics from the same contour-category table used for Hill diversity estimation. These included the proportion of singleton contour categories, the proportion of repeated contour categories, the proportional abundance of the most common contour category, and ARTwarp category evenness. Because individual callers could not be assigned to whistles, these metrics were interpreted as operational measures of contour recurrence and stereotypy rather than confirmed signature- or non-signature-whistle classifications.

Acoustic composition metrics were derived from pairwise whistle dissimilarities generated in Luscinia, which employs a fuzzy-comparison framework based on DTW Euclidean distances to compare fundamental frequency contours.^55^^,^^57^ Pairwise acoustic distances were then computed using DTW with parameter settings following previously published Luscinia-based analyses of dolphin whistle contours.^55^^,^^57^^,^^58^ These settings emphasize fundamental-frequency contour shape and local frequency modulation while allowing temporal differences to be accommodated through DTW so that whistles with similar spectral trajectories but differences in duration to be compared directly. The following settings were used for DTW: DTW: Time = 10; Fundamental frequency = 1; Fundamental frequency change = 1; Vibrato amplitude = 0.25; all other weightings = 0; Compression factor = 0.001; Minimum element length = 10; Time SD weighting = 1; Non-time SD weighting = 0; ArcTan = 0; Syllable repetition = 0; Maximum warp = 100%; Cost for stitching and alignment error = 0; Selected Weight by relative amplitude, Weight features with SDs, Log transform frequencies, Interpolate in TW, and Dynamic warping; Alignment Points = 5 points. Under this configuration, the resulting dissimilarity score for each whistle pair reflects the degree of acoustic divergence in spectral–temporal contour structure, integrating differences in contour geometry, frequency modulation, vibrato patterns, and local shape features. Lower distances indicate highly similar whistle contours, while higher distances represent greater divergence. This continuous, fuzzy comparison approach captures graded variation among whistles and avoids imposing categorical whistle-type boundaries, a feature particularly well suited to flexible, non-discrete dolphin vocal systems.

The complete Luscinia-derived dissimilarity matrix was exported, and all subsequent analyses were performed in R. For each sighting, we calculated group-level summaries of acoustic composition based on the distribution of pairwise distances, including the mean, standard deviation (SD), coefficient of variation (CV), and range of distances. These metrics describe the central tendency, dispersion, and heterogeneity of whistle structure within each group, providing a continuous multivariate representation of acoustic organization and within-group repertoire diversification.

### Quantification and statistical analysis

#### Data filtering and inclusion criteria

To ensure robustness and comparability across groups, only groups meeting the following inclusion criteria were retained.

1. ≥60% of individuals were positively identified with photoID.
2. No singletons (individuals sighted only once) were present; and
3. Each group had ≥2 whistles per identified individual (i.e., ≥2× group size total whistles).

After filtering, 37 group-level sighting events were retained for social-network and acoustic repertoire analyses.

#### Elastic-net regression

All analyses were conducted in R 4.3.3.^81^ Relationships between social network structure and acoustic diversity or composition were examined using a multivariate Gaussian elastic-net regression in the glmnet package to address issues with numerous and correlated predictors.^82^ Predictor variables included predominant behavioral state, group size, number of calves/juveniles, and group-level social network metrics: mean bond strength, SD bond strength, CV bond strength, group clustering coefficient, network density, and degree centralization. Predominant behavioral state was entered categorically, with foraging as the reference level. All predictors were penalized equally, allowing behavioral, demographic, and social-network variables to compete within the same regularization framework. Response variables were analyzed in three acoustic blocks: categorical repertoire diversity metrics based on Hill numbers, continuous acoustic distance metrics derived from pairwise DTW comparisons, and supplementary ARTwarp recurrence/stereotypy metrics. Elastic net was chosen because the social-network predictors quantify related dimensions of group structure and may exhibit covariance, which can lead to unstable coefficient estimates in conventional multiple-regression frameworks. The elastic net penalty combines L1 (lasso) and L2 (ridge) regularization, allowing it to simultaneously perform variable selection, handle multicollinearity, and provide more reliable coefficient estimates in the presence of correlated predictors. Models used an elastic-net mixing parameter of α = 0.5, providing equal contribution of L1 (lasso) and L2 (ridge) penalties.

Models were fitted using 10-fold cross-validation to select the optimal regularization parameter (λ_min) and elastic-net coefficients were extracted to evaluate the magnitude and direction of social predictors. Response variables were z-standardized prior to fitting, and predictor variables were standardized internally by glmnet during model fitting. Model validation included inspection of residual distributions, and predictors were assessed for strong collinearity prior to model fitting (all pairwise correlations and VIF values were low; VIF <2). Coefficients equal to zero indicate predictors not retained after regularization. Because elastic net is used for variable selection and shrinkage rather than null-hypothesis testing, results are interpreted in terms of coefficient retention, direction, and relative magnitude rather than *p*-values.

#### Non-metric multidimensional scaling

To visualize multivariate patterns in whistle similarity, non-metric multidimensional scaling (NMDS) was performed on the Luscinia DTW dissimilarity matrix using metaMDS in the vegan package. The ordination was fitted in two dimensions (k = 2) with a maximum of 100 random starts and no autotransformation. Ordination fit was evaluated using NMDS stress. For visualization, sightings were divided into low, medium, and high clustering terciles based on group clustering coefficient, and sighting-level centroids and 95% confidence ellipses were plotted within the resulting ordination space.

### Additional resources

This study did not generate additional resources.
