# Supplementary material for: Communicative diversity scales with social network structure in bottlenose dolphins
Source: iScience. 2026 Oct 20;29(11):117954. doi: 10.1016/j.isci.2026.117954 (PMC13697327; doi:10.1016/j.isci.2026.117954)
Supplement: Supplementary file 1 [file mmc1.pdf]

**iScience, Volume *29***

## Supplemental information

# Communicative diversity scales with social network structure in bottlenose dolphins

**Maia Austin and Laura J. May-Collado**

**<u>Supplementary Information</u>**

## Supplemental figures and legends

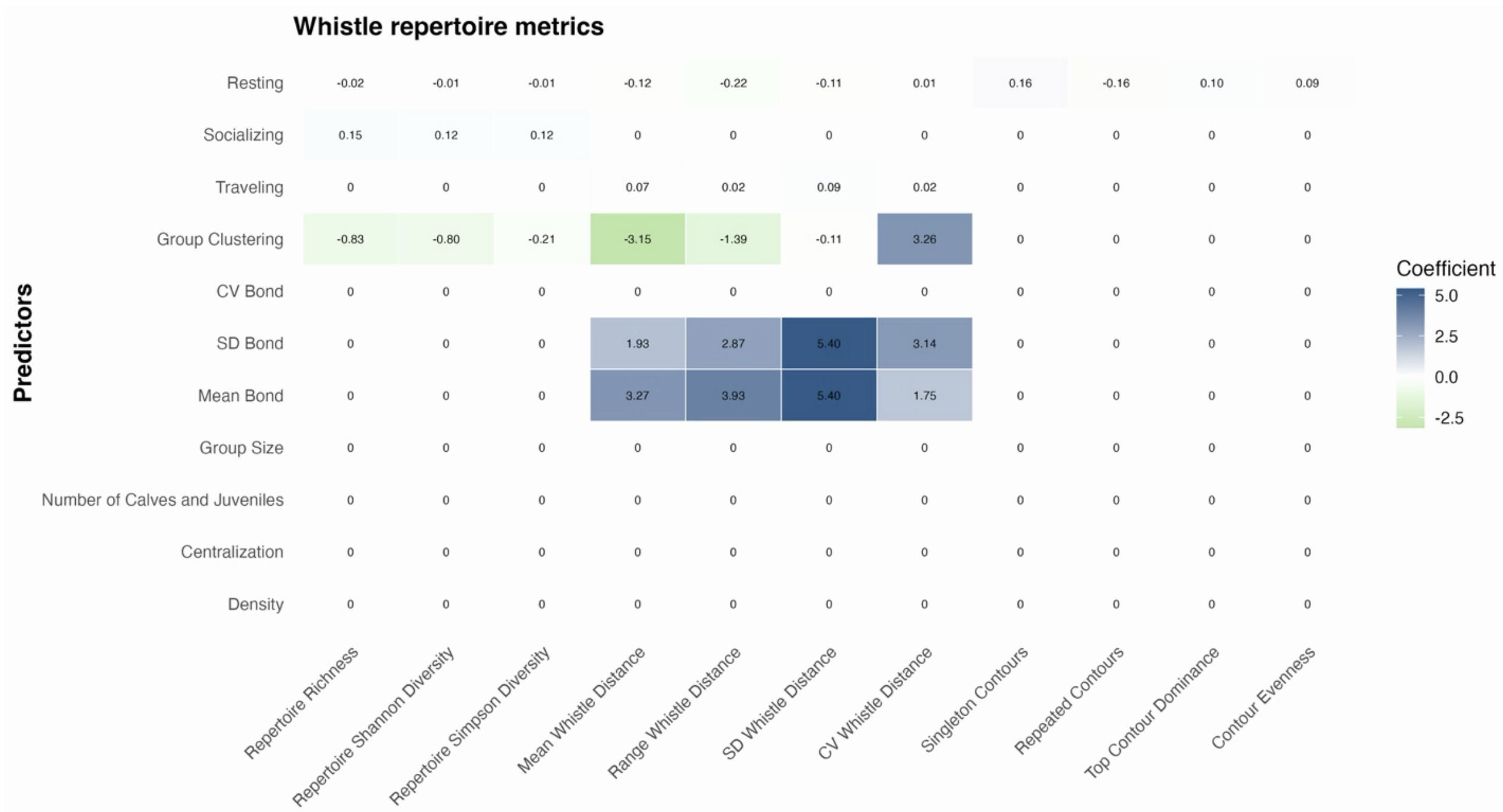


Figure S1. Full elastic-net coefficient heatmap including supplementary recurrence/stereotypy responses. Standardized coefficients from equal-penalty elastic-net models predicting whistle repertoire metrics from predominant behavior, group size, calf/juvenile count, and group-level social network metrics. Response variables include primary repertoire diversity metrics, acoustic-distance metrics, and supplementary ARTwarp recurrence/stereotypy metrics. Recurrence metrics include the proportion of singleton contour categories, proportion of repeated contour categories, dominance of the most common contour category, and contour evenness. Because individual callers could not be assigned to whistles, recurrence metrics were interpreted as operational proxies for contour recurrence and stereotypy rather than confirmed signature- or non-signature-whistle classifications. White cells indicate coefficients equal to zero after regularization; colored cells indicate retained standardized coefficients at the cross-validated λmin. The recurrence/stereotypy responses showed limited coefficient retention relative to the primary diversity and acoustic-distance outcomes.

**Supplemental tables**

Supplementary Table 1 – Group-level social network metrics for each sighting included in the final analysis. Values summarize encounter-level social structure derived from association networks, including group size, number of calves and juveniles present, mean bond strength, heterogeneity of association strengths (SD and CV of bond strength), network density, group clustering coefficient, and degree centralization.

| Group | Calves + Juveniles | Group Size | Mean Bond | SD Bond | CV Bond | Density | Clustering | Central-ization |
|---|---|---|---|---|---|---|---|---|
| 1 | 2 | 5 | 0.34 | 0.17 | 0.49 | 2.63 | 0.48 | 4.17 |
| 2 | 0 | 4 | 0.35 | 0.18 | 0.52 | 6.97 | 0.52 | 8.17 |
| 3 | 2 | 4 | 0.37 | 0.20 | 0.54 | 4.71 | 0.51 | 3.00 |
| 4 | 3 | 1 | 0.36 | 0.18 | 0.50 | 5.76 | 0.50 | 4.25 |
| 5 | 2 | 2 | 0.37 | 0.19 | 0.51 | 6.04 | 0.50 | 3.92 |
| 6 | 0 | 6 | 0.33 | 0.18 | 0.53 | 2.71 | 0.50 | 2.31 |
| 7 | 0 | 9 | 0.34 | 0.18 | 0.52 | 2.37 | 0.52 | 1.98 |
| 8 | 0 | 4 | 0.35 | 0.19 | 0.54 | 4.58 | 0.61 | 6.58 |
| 9 | 0 | 9 | 0.33 | 0.17 | 0.51 | 1.41 | 0.58 | 1.68 |
| 10 | 0 | 6 | 0.32 | 0.16 | 0.49 | 3.38 | 0.61 | 4.50 |
| 11 | 0 | 4 | 0.37 | 0.19 | 0.50 | 5.05 | 0.57 | 6.25 |
| 12 | 0 | 4 | 0.35 | 0.17 | 0.50 | 2.59 | 0.58 | 6.85 |
| 13 | 0 | 13 | 0.34 | 0.18 | 0.51 | 1.16 | 0.58 | 1.11 |
| 14 | 0 | 3 | 0.35 | 0.19 | 0.55 | 8.11 | 0.60 | 29.50 |
| 15 | 1 | 4 | 0.32 | 0.13 | 0.41 | 4.60 | 0.68 | 43.00 |
| 16 | 4 | 2 | 0.37 | 0.20 | 0.54 | 3.25 | 0.58 | 3.40 |
| 17 | 3 | 3 | 0.35 | 0.17 | 0.49 | 3.40 | 0.56 | 2.80 |
| 18 | 0 | 6 | 0.37 | 0.19 | 0.51 | 4.95 | 0.47 | 2.35 |
| 19 | 1 | 6 | 0.36 | 0.18 | 0.50 | 3.76 | 0.45 | 4.65 |
| 20 | 1 | 5 | 0.36 | 0.19 | 0.52 | 3.78 | 0.58 | 4.60 |
| 21 | 1 | 7 | 0.34 | 0.18 | 0.52 | 1.65 | 0.54 | 2.43 |
| 22 | 1 | 4 | 0.37 | 0.20 | 0.54 | 6.22 | 0.54 | 11.50 |
| 23 | 1 | 6 | 0.35 | 0.17 | 0.49 | 3.15 | 0.61 | 5.65 |
| 24 | 1 | 4 | 0.35 | 0.17 | 0.50 | 3.13 | 0.66 | 5.70 |
| 25 | 1 | 2 | 0.36 | 0.21 | 0.57 | 11.61 | 0.53 | 16.50 |
| 26 | 2 | 8 | 0.33 | 0.17 | 0.50 | 1.62 | 0.64 | 2.44 |
| 27 | 1 | 4 | 0.36 | 0.19 | 0.53 | 4.20 | 0.56 | 3.70 |
| 28 | 0 | 3 | 0.30 | 0.12 | 0.42 | 6.01 | 0.60 | 34.00 |
| 29 | 1 | 6 | 0.38 | 0.19 | 0.51 | 3.40 | 0.56 | 5.65 |
| 30 | 1 | 2 | 0.41 | 0.19 | 0.47 | 9.20 | 0.61 | 30.50 |

Supplementary Table 2 – Group-level acoustic repertoire metrics for each sighting included in the final analysis. Reported values include Hill-number diversity indices (richness $q = 0$, Shannon diversity $q = 1$, Simpson diversity $q = 2$) derived from ARTwarp whistle categories, as well as summary statistics of within-group acoustic dissimilarity (mean, median, minimum, maximum, standard deviation, coefficient of variation, and range of whistle distances).

| **Group** | **Richness** | **Shannon** | **Simpson** | **Mean Dist.** | **Med. Dist.** | **Max. Dist.** | **Min. Dist.** | **SD Dist.** | **CV Dist.** | **Range Dist.** |
|---|---|---|---|---|---|---|---|---|---|---|
| 1 | 173.82 | 88.24 | 28.43 | 0.41 | 0.38 | 0.97 | 0.02 | 0.21 | 0.51 | 0.95 |
| 2 | 66.03 | 69.70 | 71.52 | 0.39 | 0.36 | 0.84 | 0.02 | 0.18 | 0.47 | 0.81 |
| 3 | 295.61 | 237.79 | 121.98 | 0.41 | 0.39 | 1.00 | 0.00 | 0.20 | 0.49 | 1.00 |
| 4 | 254.79 | 209.21 | 150.69 | 0.41 | 0.37 | 1.00 | 0.00 | 0.20 | 0.48 | 1.00 |
| 5 | 266.34 | 213.86 | 146.28 | 0.37 | 0.34 | 1.00 | 0.00 | 0.19 | 0.51 | 1.00 |
| 6 | 284.00 | 232.66 | 172.93 | 0.39 | 0.35 | 1.00 | 0.00 | 0.19 | 0.50 | 1.00 |
| 7 | 311.85 | 306.04 | 260.29 | 0.42 | 0.38 | 0.95 | 0.01 | 0.20 | 0.49 | 0.94 |
| 8 | 221.56 | 197.28 | 143.02 | 0.44 | 0.40 | 1.00 | 0.00 | 0.24 | 0.54 | 1.00 |
| 9 | 114.49 | 120.74 | 116.12 | 0.39 | 0.36 | 0.96 | 0.02 | 0.21 | 0.52 | 0.95 |
| 10 | 87.52 | 83.68 | 66.58 | 0.41 | 0.36 | 0.93 | 0.00 | 0.24 | 0.57 | 0.92 |
| 11 | 65.70 | 38.37 | 18.21 | 0.33 | 0.27 | 0.97 | 0.00 | 0.22 | 0.66 | 0.97 |
| 12 | 89.10 | 63.20 | 32.06 | 0.36 | 0.32 | 0.91 | 0.01 | 0.20 | 0.55 | 0.90 |
| 13 | 276.63 | 232.71 | 182.35 | 0.32 | 0.29 | 1.00 | 0.00 | 0.17 | 0.52 | 1.00 |
| 14 | 80.11 | 71.12 | 52.85 | 0.36 | 0.34 | 0.99 | 0.01 | 0.22 | 0.61 | 0.98 |
| 15 | 167.47 | 148.82 | 66.18 | 0.33 | 0.32 | 0.90 | 0.02 | 0.17 | 0.53 | 0.87 |
| 16 | 204.12 | 135.46 | 83.12 | 0.38 | 0.32 | 1.00 | 0.00 | 0.23 | 0.61 | 1.00 |
| 17 | 86.76 | 90.47 | 88.02 | 0.37 | 0.34 | 0.83 | 0.04 | 0.17 | 0.45 | 0.79 |
| 18 | 245.55 | 169.10 | 102.06 | 0.36 | 0.31 | 1.00 | 0.00 | 0.21 | 0.58 | 1.00 |
| 19 | 172.80 | 175.42 | 156.83 | 0.48 | 0.45 | 0.99 | 0.00 | 0.25 | 0.53 | 0.99 |
| 20 | 83.45 | 79.27 | 64.89 | 0.33 | 0.29 | 0.84 | 0.00 | 0.21 | 0.62 | 0.84 |
| 21 | 260.97 | 247.88 | 196.67 | 0.51 | 0.52 | 0.95 | 0.01 | 0.23 | 0.44 | 0.94 |
| 22 | 242.02 | 184.23 | 115.10 | 0.43 | 0.40 | 1.00 | 0.00 | 0.22 | 0.51 | 1.00 |
| 23 | 225.99 | 165.28 | 94.36 | 0.35 | 0.33 | 1.00 | 0.00 | 0.18 | 0.51 | 1.00 |
| 24 | 95.04 | 49.12 | 27.34 | 0.28 | 0.19 | 0.96 | 0.00 | 0.22 | 0.80 | 0.96 |
| 25 | 146.34 | 147.26 | 128.66 | 0.40 | 0.35 | 1.00 | 0.00 | 0.21 | 0.53 | 1.00 |
| 26 | 89.00 | 69.13 | 39.02 | 0.35 | 0.31 | 0.98 | 0.00 | 0.20 | 0.59 | 0.98 |
| 27 | 34.21 | 30.35 | 22.26 | 0.37 | 0.30 | 0.89 | 0.02 | 0.26 | 0.69 | 0.87 |
| 28 | 229.79 | 164.99 | 57.99 | 0.36 | 0.33 | 0.96 | 0.01 | 0.17 | 0.47 | 0.95 |
| 29 | 43.80 | 34.70 | 24.90 | 0.40 | 0.42 | 0.91 | 0.00 | 0.20 | 0.49 | 0.91 |
| 30 | 224.49 | 157.17 | 50.16 | 0.48 | 0.43 | 1.00 | 0.02 | 0.25 | 0.52 | 0.98 |
